# The associations of hostility and defensiveness with telomere length are influenced by sex and health status

**DOI:** 10.1186/s13293-020-00349-w

**Published:** 2021-01-04

**Authors:** Louisia Starnino, Gilles Dupuis, Lambert Busque, Vincent Bourgoin, Marie-Pierre Dubé, David Busseuil, Bianca D’Antono

**Affiliations:** 1 Research Center, Montreal Heart Institute, 5000 Rue Bélanger, Montréal, QC H1T 1C8 Canada; 2 Department of Psychology, Université du Québec à Montréal, Montreal, Canada; 3 Research Center, Hematology Division, Hôpital Maisonneuve-Rosemont, Université de Montréal, Montreal, Canada; 4 Department of Psychology, Université de Montréal Pavillon Marie-Victorin, Montreal, Canada

**Keywords:** Telomere length, Hostility, Defensiveness, Cardiovascular disease, Sex and age

## Abstract

**Background:**

Shorter telomere length (TL) may indicate premature cellular aging and increased risk for disease. While there is substantial evidence for shorter TL in individuals suffering from psychiatric disorders, data is scarce on maladaptive personality traits related to coronary artery disease (CAD). The purpose of this study was to evaluate the association of TL with hostility and defensiveness in individuals with CAD or other non-cardiovascular illnesses and whether associations were moderated by CAD status and sex.

**Methods:**

One thousand thirty-six individuals (*M*_age_ = 65.40 ± 6.73 years) with and without CAD completed the Marlowe-Crowne Social Desirability Scale and the Cook–Medley Hostility Scale. Relative TL was measured via quantitative polymerase chain reaction of total genomic DNA samples. Analyses involved hierarchical regressions on TL, performed separately for hostility and defensiveness, controlling for pertinent sociodemographic, behavioural, and medical risk factors. Separate analyses were performed on 25 healthy participants.

**Results:**

A hostility by sex interaction emerged (*β* = − .08, *p* = .006) in the patient groups, where greater hostility was associated with shorter TL in women only (*p* < .01). A Defensiveness by CAD status interaction (*β* = − .06, *p* = .049) revealed longer TL in more defensive CAD patients only (*p* = .06). In healthy men, shorter TL was observed in those with greater defensiveness (*β* = .52, *p* = .006) but lower hostility (*β* = − .43, *p* = .049).

**Conclusion:**

Hostility and defensiveness are differentially associated with TL as a function of sex and health status. The implication of these results for health remains to be determined, but propose an additional pathway through which the effect of maladaptive personality traits may contribute to CV and other disease.

## Introduction

Hostility is a stable personality trait accompanied by frequent feelings of anger, aggressive behaviour, and/or the tendency to devalue or to be cynical of others [1]. It has been established as a robust risk factor for the development of coronary artery disease (CAD) and premature mortality [2, 3] as well as with all-cause mortality [4]. Defensiveness, a personality trait characterized by the tendency to present oneself in a favourable manner [5], has similarly been associated with an increased risk for CAD and mortality [6, 7]. In defensiveness, individuals deny, avoid, or repress personal information (e.g., physical symptoms, traits, behaviours, negative affect) perceived as non-desirable in order to protect one’s vulnerable self-esteem and/or to maintain socials bonds [8, 9].

The biological mechanisms responsible for the ill effects of hostility and defensiveness remain to this day poorly understood. It is hypothesized, however, to at least partly reflect the sustained or cumulative impact of alterations in various intermediary risk factors for CAD (e.g., physiological reactivity to stress, inflammation, blood pressure, and other metabolic parameters) observed in more hostile or defensive individuals [10–16]. Telomere regulation may also be impacted. Telomeres are specialized DNA-protein-repetitive sequences that cap eukaryotic chromosome ends during cellular division in order to prevent end-to-end recombination, deterioration, or fusion with neighbouring chromosomes and as such, play an integral role in preventing the loss of genetic data [17]. Telomere length (TL) shortens with every cell division, reflecting the cellular aging of organisms. Shortening depends on several factors, such as the rate of cellular division and levels of telomerase, a ribonucleoprotein reverse transcriptase enzyme that adds telomeric DNA [18].

CAD patients have significantly shorter leukocyte TL than their age-matched non-CVD counterparts [19]. While conflicting data exist, shorter TL has been associated with increased incidence for CAD events, CAD progression, and increased mortality from CAD [20–22]. Individuals at risk for CAD by virtue of metabolic dysregulations [23], hypertension [24], diabetes [25], and smoking [26] have also been shown to possess shorter TL. As such, TL may be a potential biomarker for CVD risk and prognosis [20].

While significantly shorter TL have been found in stressed (*r* = − 0.06) [27] or depressed (*r* = − 0.21) [28] individuals in comparison with psychologically healthier individuals, research examining stable personality traits has been scarce [29–32] and has led to conflicting results. For example, in a moderate-sized cross-sectional study of older women and men without CVD, Brydon and colleagues [29] found that men (but not women) with greater cynical hostility had shorter TL compared to those less hostile (*β* = − 0.25, *p* = 0.001), independently of age, grade of employment, body mass index (BMI), and waist circumference. In a sample of predominantly male U.S. military veterans suffering from various chronic medical conditions, those with shorter TL (based on relative TL < 1), reported greater levels of hostility compared to those with longer TL [31]. In contrast, we recently reported that greater hostility (and increases in hostility over a 3-year period) was associated with longer (not shorter) TL in healthy men and women [30]. At this time, it remains unclear whether hostility is reliably associated with TL. Only one study examined whether TL differs as a function of defensiveness and found greater defensiveness was associated with shorter TL in healthy men and women [30].

While data regarding potential sex differences in the relations between hostility, defensiveness, and TL is scarce and mixed, men consistently show shorter TL [33, 34] and are more likely to harbour and express hostile attitudes and behaviour compared to women [35]. They may also be at greater risk for cardiovascular events from hostility compared to women [3]. On the other hand, differential biological mechanisms and socialization of women versus men may contribute to greater concern for interpersonal relationships [36, 37] and propensity to present a favourable impression in women versus men [38] and hence to differential relations between defensiveness and TL in men versus women. In support of this, we have previously reported that in healthy individuals aged 20–64 years, higher levels of defensiveness were associated with greater cardiovascular stress reactivity [12] and a worse metabolic profile [13] in women but not men.

In addition, while differences in TL have been observed as a function of CAD status, it remains to be explored whether the relation between personality and TL also differs as a function of the health status of individuals.

The current study sought to confirm whether TL were associated with hostility and defensiveness in a large sample of older men and women with CAD, other non-CVD illness, or a healthy profile and whether these relations were influenced by sex and health status.

It was hypothesized that hostility and defensiveness would be associated with TL. However, given the limited and mixed findings for hostility and absence of studies among patient populations, no hypotheses were elaborated regarding the direction or magnitude of the relations nor any influence of sex or health status on the results.

## Methods

### Description of cohort and sample selection

This study is part of an ongoing prospective research project (BEL-AGE) on psychological burden and premature aging. It was approved by the ethics committee of the Montreal Heart Institute. Recruitment for this study began in September 2012 and finished in May 2017.

At the time of preparing this manuscript, data were available for 1121 men (*n* = 719) and women (*n* = 402), 34–76 years of age (*M*_age_ = 65.1 ± 7.07). Participants were recruited from the André and France Desmarais Hospital Cohort of the Montreal Heart Institute. Any person working at or attending the hospital for any reason were eligible for the Montreal Heart Institute (MHI) Cohort [39].

Eligibility criteria for BEL-AGE were determined as follows: at entry in the MHI Cohort, (a) aged between 30 and 70 years (for reasons of feasibility of recruitment and follow-up), (b) living in the greater Montreal area, (c) speaks French or English, (d) no previous or current diagnosis of major cognitive impairment or serious psychological disorders (e.g. bipolar disorder, schizophrenia, delirium, or dementia as reported by the patient and/or medical files) that would prevent understanding or participating in all aspects of the study, (e) no previous or current diagnosis of other major life-threatening diseases (e.g., Creutzfeldt-Jakob disease, amyotrophic lateral sclerosis, AIDS, cancer), (f) not currently pregnant or breast feeding, and (g) no family member (including spouse) already participating or scheduled to participate in the study. Skin cancer (*n* = 60) was not excluded given its high prevalence and benign course when diagnosed early. Presence of CAD was defined by the experience of a previous myocardial infarction, coronary artery bypass, coronary angioplasty, or stenosis more than 50% on an angiography. Non-CVD status was defined by the absence of CAD, angina, arrhythmia, congenital heart disease, heart failure, cardiomyopathy, and stroke. To ensure they met these criteria, individuals were interviewed by phone before being invited to participate and a medical chart review was performed to ascertain group (CAD vs. non-CVD) membership. The types of conditions experienced by the non-CVD illness group were diverse (e.g., arthritis, diabetes, hypertension, gastrointestinal diseases, hypercholesterolemia, and asthma). While we had initially sought to recruit a larger sample of older individuals with no significant health issue, we were unable to secure a significant number of healthy participants within the population from which we recruited despite extended recruitment. Only 25 individuals suffered from no notable medical condition (very healthy group).

Data for the article were obtained during the BEL-AGE evaluation performed 4.88 (± 0.76) years after participants’ entry into the MHI cohort.

### Procedure

Participants were scheduled for an interview between 8:00 and 10:00 in the morning on a weekday to control for circadian rhythm. They were asked to abstain from drinking (with the exception of water), smoking, and exercise for 12 h before their scheduled appointment. They were also asked to refrain from the use of drugs or alcohol for the 24 h preceding their appointment. Prescribed medication was permitted. Participants were sent home, and their appointment rescheduled if they did not adhere to these instructions or if they were presenting with symptoms of flu. Research assistants were trained to maintain a neutral tone and expression during testing. Once participants provided written consent, anthropomorphic data (weight, height, and waist circumference) and 35 mL of blood were obtained. Participants completed sociodemographic, health behaviour, and psychological questionnaires.

### Outcome measures

#### Hostility

The short form of the Cook–Medley Hostility Inventory (CMHo-T) [40], consisting of 39 true-or-false items, was used to measure cynicism, hostile affect, and aggressive responding [41]. The short form’s internal consistency (*α* = 0.85) [42] and test-retest reliability (*r* = 0.74) are both good [43]. In the current sample, the internal consistency was 0.81 while the 5-year test-retest reliability was 0.84.

#### Defensiveness

The short form of The Marlowe-Crowne Social Desirability scale (MCSD) [5, 44], consisting of 14 true or false items, was used to assess whether respondents are answering in a culturally sanctioned and desirable manner. The items present behaviours that are desirable but infrequent (e.g., “I’m always willing to admit it when I make a mistake”) and behaviours that are undesirable but frequent (e.g., “I like to gossip at times”). The short-form MCSD has been found to have adequate psychometric properties, with internal consistency estimates of 0.62–0.77 [44] and a test-retest correlation of 0.74 [45]. In the current sample, the internal consistency was 0.68 and the test-retest reliability was 0.78. The MCSD scale has been shown to be a valid measure of the defensiveness construct [46, 47] and has been frequently used to assess defensiveness in the area of cardiovascular health psychology.

#### Telomere length

DNA was extracted from peripheral blood leukocytes using standard methods, and TL was measured by quantitative polymerase chain reaction (qPCR) using the modified method [48] of the protocol described by Cawthon [49]. The DNA extraction was done automatically on the BioRobot M48 system (Qiagen). The buffy coat samples are thawed at room temperature. For each sample, 55 μL of cells are mixed with 95 μL of RPMI-1640 (Invitrogen, # 11875-093). Extraction on the BioRobot M48 is carried out using the MagAttract DNA Blood M48 kit (Qiagen, # 951356). DNA is diluted in 200 μL of RNase-free water. DNA is stored at − 80 °C and quantified by UV spectrophotometer (Tecan, Infinite M1000 Pro) by measuring the specific UV absorbance of nucleic acids at 260 nm.

This modified method for TL [48, 49] is based on determining the number of telomeric repeat sequence (T) on the number of copies of a single gene (*RPLP0*, 60S acidic ribosomal protein P0) (S). A T/S ratio was calculated for each sample of DNA tested. A relative ratio was obtained by comparing the ratio T/S of a sample to the ratio T/S of a reference DNA sample (obtained from a single individual and used to generate the standard curves) giving a value T/S of 1. Therefore, the relative ratio of a sample represents the number of copies of telomeres relative to the reference sample. For each DNA sample, T and S qPCR SYBR® green reactions are assembled with the epMotion 5075 TMX (Eppendorf) automated pipetting systems. Each 20 μL reaction contained 7.5 ng of DNA, 10 μL of QuantiFast SYBR® Green PCR Master Mix (Qiagen, # 204057), and 450 nM of primers Tel1b (CGGTTTGTTTGGGTTTGGGTTTGGGTTTGGGTTTGGGTT) and Tel2b (GGCTTGCCTTACCCTTACCCTTACCCTTACCCTTACCCT) for the T amplification or 300 nM of the primer 36B4u (CAGCAAGTGGGAAGGTGTAATCC) and 500 nM of the primer 36B4d (CCCATTCTATCATCAACGGGTACAA) for the S amplification. Serial dilutions of the reference DNA are made (25 to 1.562 ng/μL) for each 96-well plate, and the values obtained were used to establish a standard curve in order to calculate the T and S values of the tested samples. All qPCR were performed on the ViiA™7 real-time PCR system (Applied Biosystems) and with the QuantStudio analysis software (Applied Biosystems). The thermal amplification profile of telomeres (T) is described as follows: 5 min at 95 °C, followed by 25 cycles of 95 °C for 10 s and 56 °C for 1 min. The thermal amplification profile of the single- copy gene (S) is described as follows: 5 min at 95 °C followed by 40 cycles of 95 °C for 10 s and 60 °C for 40 s. All samples were measured in triplicate, and their mean was used for analyses.

### Sociodemographic and health confounders

Sociodemographic (sex, age, ethnicity, years of schooling, marital status, personal and family income), and personal and family medical history were collected via interview. Data on behavioural risk factors (tobacco, alcohol, caffeine consumption, diet, and physical activity), weight, height, and waist circumference were also obtained.

### Inflammatory and metabolic confounders

The blood samples were frozen (− 80 °C) and then assayed in batch. C-Reactive protein (CRP) was measured from plasma using the Siemens CardioPhase hsCRP assay on the BN ProSpec Nephelometer (Siemens Healthcare Diagnostics Products GmbH, Marburd, Germany). The minimal detectable hsCRP concentration was 0.18 mg/L. Lipids and glucose were assayed using respective reagent Flex on the multianalyzer Dimension RxL Max (Dade Behring Diagnostics, Marburg, Germany) with heparinized plasma. To measure waist circumference (WC), the participant’s waistline was exposed and the bottom of a measuring tape was aligned with the top of the hip bone and stretched across the midsection over the navel [50]. Blood pressure was obtained during a 5-min continuous reading at rest using a Finometer (Finapres Finometer, Amsterdam, the Netherlands) and analyzed offline in LabChart (ADInstruments, Oxford, UK). The mean arterial pressure was used for statistical analyses.

### Statistical analyses

Analyses were performed on participants with complete sociodemographic, behavioural, telomere, biochemical, and psychological data (*n* = 1061).

#### Preliminary analyses

No significant sociodemographic, psychological or physiological differences were observed in participants with (*n* = 1061) or without (*n* = 58) complete data.TL was positively skewed and a natural logarithm transformation was applied to ensure normal distribution.

Potential covariates were based on the TL literature [51], as well as on the results of bivariate correlations with TL. Only covariates showing an association *p* < 0.10 with TL in our sample were retained. These included age, sex, employment status, waist circumference, number of cigarettes per day, hours of exercise/weekly, alcoholic beverages/weekly, mean arterial pressure, total cholesterol, and glucose, as well as history of hypertension, diabetes, and/or dyslipidemia. To identify any differences between CAD and non-CVD groups, independent samples *T* test (for continuous variables in unequal sample sizes) and chi-squares (for categorical variables) were used on demographic measures. Welch’s ANOVAs were performed to examine whether TL, CMHo-t, and MCSD differed as a function of CAD status and sex of participants. Effect sizes (Cohen’s *d*) were calculated via a statistical tool by Lenhard and Lenhard [52].

#### Multivariate associations of personality with TL and moderating influences of CAD status and/or sex among the patient groups

Relationships between psychological traits and TL in the patient groups were analyzed using hierarchical linear regression analyses, performed separately for hostility and defensiveness. Covariates and the potential moderators, sex, and CAD status were forced into Block 1. The psychological trait was entered into Block 2, while its possible two- and three-way interactions with CAD status and/or sex were entered stepwise in Block 3. Interactions were formed from centred variables.

Data were analyzed using the IBM SPSS Statistics 24.0 software (IBM Corporation, Somers, NY, USA), and moderation analyses were performed using the computational tool PROCESS version 3.4 [53]. A two-sided *p* value < 0.05 was considered statistically significant for main effects. However, given reduced power to detect significant interactions, the latter were explored when they met a *p* value < 0.10 to minimize type II error [54, 55]. Simple slope analyses were performed on values ± 1 SD for hostility and defensiveness. Interactions were created from centred variables.

#### Analyses in healthy individuals

Given the much smaller sample size of the healthy group and their vastly differing characteristics, analyses were performed separately for this group. The standardized residual of TL (controlling for age, BMI, employment status, and years of schooling) was used as the dependent variable. Sex, personality trait, and their interaction were included as predictors in each regression.

## Results

Table 1 presents participant characteristics. Individuals with CAD had significantly more sociodemographic, behavioural, and medical risk factors for CVD than their non-CVD counterparts. Men showed a worse CV profile than women (*p* < .001), but did not differ on other demographic variables. In the non-CVD illness group only, men were more likely to have a history of HTA, hypercholesterolemia, and diabetes compared to women (all *p*’s < .001).

**Table 1:** Participant characteristics

	CAD	Non-CVD	CAD and non-CVD combined	Healthy
	*n* = 598	*n* = 438	*n* = 1036	*n* = 25
***Demographic variables, n (%)*** **or** ***M*** **(± SD); (range)**				
Age (years)**	66.13 (± 6.25); [40–71, 73–77]	64.41 (± 7.23); [35–71, 73–77]	65.40 (± 6.73); [35–71, 73–77]	55.68 (± 0.19); [34–70]
Sex, *n* (%) **				
Men	482 (80.60)	193 (44.10)	675 (65.20)	14 (56)
Caucasian, *n* (%)	593 (99.20)	428 (97.77)	1 021 (98.60)	24 (96)
Years of schooling**	13.95 (3.76)	14.86 (3.58)	14.33 (3.71)	15.56 (3.66)
Currently employed, *n* (%)**	135 (22.60)	141 (32.20)	276 (26.60)	14 (56)
Civil status, *n* (%)				
Married/living with someone	427 (71.40)	326 (74.50)	753 (72.70)	21 (84)
Single	48 (8)	47 (10.70)	95 (9.20)	2 (8)
Divorced, separated, or widowed	123 (20.60)	65 (14.88)	188 (18.20)	2 (8)
Annual family income, *n* (%)				
≤ $29,999	86 (14.40)	27 (6.20)	113 (10.90)	2 (8)
$30,000–59,99	206 (34.40)	154 (35.20)	360 (34.70)	3 (12)
≥ $60,000**	306 (51.17)	258 (58.90)	564 (54.33)	20 (80)
***Behavioural variables, n (%)*** **or M (± SD)**				
Smoker, *n* (%)**	81 (13.50)	25 (5.70)	106 (10.20)	2 (8)
Hours of exercise/week, *M* (± SD)	2.85 (± 3.29)	3.31 (± 3.39)	3.04 (± 3.34)	3.07 (± 3.38)
Glasses of alcohol/week, *M* (± SD)	6.47 (± 8.14)	5.70 (± 6.57)	6.12 (± 7.51)	4.96 (± 5.01)
***Physiological variables, M*** **(± SD)**				
Telomere length (T/S ratio)***	0.83 (± 0.18); [0.44–1.5]	0.89 (± 0.19); [0.48–1.68]	0.86 (± 0.19); [0.44–1.68]	0.94 (± 0.15); [0.72-1.26]
Body mass index (kg/m^2^)**	29.80 (± 5.11)	28.59 (± 4.99)	29.28 (± 5.10)	27.15 (± 2.83)
Waist circumference (cm)**	102.96 (± 13.47)	96.42 (± 13.78)	100.18 (± 13.98)	90.64 (± 10.91)
Mean arterial pressure (mmHg)**	89.88 (± 13.85)	95.90 (± 14.54)	92.36 (± 14.41)	97.42 (± 14.38)
Glucose (mmol/L)**	6.46 (± 1.53)	5.89 (± .97)	6.22 (± 1.36)	5.64 (± 1.03)
Triglycerides (mmol/L)**	1.69 (± 0.80)	1.62 (± 0.84)	1.67 (± 0.82)	1.49 (± 0.89)
C-Reactive protein (mg/L)	2.65 (± 6.83)	2.78 (± 6.78)	2.71 (± 6.78)	2.82 (± 5.60)
***Psychological variables, M*** **(± SD)**				
Hostility*	14.39 (± 6.07)	13.27 (± 6.05)	13.91 (± 6.07)	11.72 (± 6.84)
Defensiveness	9.35 (± 2.73)	9.33 (± 2.77)	9.34 (± 2.74)	9.08 (± 2.64)
***Medical history and medication, n (%)***				
Myocardial infarction	387 (64.70)	-	-	-
Coronary artery bypass	211 (35.30)	-	-	-
Angioplasty	421 (70.40)	-	-	-
Arrhythmia	146 (24.40)	-	-	
Diabetes**	147 (24.60)	45(10.30)	192 (18.50)	-
Hypercholesterolemia **	583 (97.50)	262 (59.80)	845 (81.60)	-
Hypertension**	414 (69.20)	183 (41.80)	597 (57.61)	-
Other chronic diseases*	313 (52.34)	284 (64.55)	597 (57.51)	-
Family history of CVD**	421 (70.40)	256 (57.50)	677 (65.40)	14 (56)
Skin cancer	23 (3.80)	20 (4.60)	43 (4.20)	-
Antidepressants**	61 (10.20)	32 (7.30)	94 (9.06)	2 (8)
Prescribed cardiovascular agents**	573 (95.82)	281 (65.20)	855 (82.37)	-

*CAD* coronary artery disease patients, *non-CVD* participants with non-cardiovascular illness

Hostility = 39-item Cook–Medley Hostility Inventory; defensiveness = 14-item Marlowe-Crowne Social Desirability Scale; currently employed = includes part-time and full-time workers; other chronic diseases include osteoarthritis, autoimmune disorders, Crohn’s disease, irritable bowel syndrome; prescribed cardiovascular agents = statins, beta-blockers, calcium channel blockers, and other agents

***p < .001, **p < .01, *p < .05, ~ *p* = .07 difference between CAD and non-CVD illness group

TL was negatively associated with age in all three groups (CAD: *r* = − .22, *p <* .001; non-CVD illness: *r* = − .29, *p <* .001; healthy: *r* = − .43, *p* < .05).

### Group differences in TL, hostility, and defensiveness

Individuals with CAD had shorter TL as compared with individuals with non-CV illness (F(1,898.16) = 24.83, *p* < .001, *d* = .32). A sex main effect (F(1, 685.40) = 15.93, *p* < .001, *d* = .27) indicated shorter TL in men compared to women. As for hostility, main effects of CAD status emerged (F(1, 944.96) = 9.02, *p* < .05, *d* = .19), with greater hostility reported by individuals with CAD as compared with individuals with non-CVD illness. No group differences emerged for defensiveness. Among the healthy subsample (*n* = 25), one-way ANOVAs revealed no statistically significant sex difference in TL, hostility, and defensiveness (see Table 2 for means).

**Table 2:** Group means and Pearson correlations between TL and personality traits as a function of health status and sex

	CAD		Non-CVD		Healthy	
	Men (*n* = 482)	Women (*n* = 116)	Men (*n* = 193)	Women (*n* = 245)	Men (*n* = 14)	Women (*n* = 11)
**Mean (± SD)**						
Telomere length (T/S ratio)	0.83 (0.17)	0.86 (0.019)	0.87 (0.19)	0.90 (0.20)	0.96 (0.18)	0.91 (0.11)
Hostility	14.50 (5.18)	13.94 (5.56)	13.19 (6.26)	13.30 (5.85)	11.79 (5.79)	11.64 (8.29)
Defensiveness	9.36 (2.72)	9.32 (2.73)	9.59 (2.68)	9.13 (2.82)	9.29 (2.43)	8.82 (2.99)
**Correlations with telomere length (*r*)**						
Defensiveness	0.03	0.14	− 0.07	− 0.04	− 0.65*	0.05
Hostility	− 0.01	− 0.17	0.04	− 0.14*	0.36	− 0.39

No statistical differences between men and women emerged for all three groups

*p < .05, ~ *p* = .06; effect size: *r* = .1 (small), *r* = .3 (medium), *r* = .5 (large)

### Bivariate correlations between TL, hostility, and defensiveness

Table 2 provides the sex-specific and overall Pearson correlations between TL, hostility, and defensiveness for CAD, non-CVD, and healthy groups separately. Greater hostility was associated with shorter TL across all groups of women, though statistically significant only in the non-CVD group. The effect sizes of the correlations were within the small-to-medium range for all women, suggesting that lack of significance in the other two female groups was a function of their smaller sample size, as correlations were actually stronger than that of the non-CVD group. In healthy men, TL were longer among more hostile individuals whereas they were shorter among those who were more defensive.

### Multivariate associations of personality with TL and moderating influences of CAD status and/or sex among the patient groups (Table 3), (Fig. 1)

#### *Hostility*

A significant hostility by sex interaction emerged among the patient groups when controlling for important demographic, medical, and metabolic covariates. Simple slope analyses indicated that greater hostility was associated with shorter TL in women (*β* = − .001, *t* = − 2.82, *p* = .005), but not in men (*β* = .001, *t* = .78, *p* = .43).

**Table 3:** Results of the hierarchical regression analysis for hostility and defensiveness predicting telomere length in the patient groups

			*β*	*t*	*p*	Semipartial *r*	95% CI
**Regression 1**							
**BLOCK 1**							
Age			− 0.24	− 6.86	0.006*	− 0.21	[− 0.002, − 0.001]
Sex			0.05	1.40	0.16	0.04	[− 0.002, − 0.011]
CAD status			0.03	0.83	0.41	0.03	[− 0.004, 0.009]
Employment status			− 0.01	− 0.36	0.72	− 0.01	[− 0.008, 0.005]
#Cigarettes daily			− 0.03	− 1.13	0.26	− 0.03	[− 0.001, 0.000]
#Exercise hours/week			0.06	1.90	0.06	0.06	[0.000, − 0.001]
#Alcohol beverages/week			− 0.07	− 2.10	0.04	− 0.06	[− 0.001, − 0.002]
Waist circumference			− 0.03	− 0.99	0.32	− 0.03	[0.000, − 0.000]
Glucose			− 0.01	− 0.32	0.75	0.01	[− 0.002, 0.002]
Mean arterial pressure			0.08	2.65	0.008*	0.08	[0.000, 0.000]
Cholesterol total			0.05	1.40	0.16	0.08	[− 0.001, 0.005]
History of CVD risk			− 0.04	− 1.02	0.31	− 0.03	[− 0.014, 0.004]
**F(12,1023) = 11.08,** ***p*** **< 0.001,** ***R***^**2**^ **= 0.115,** ***R***^**2**^_**adj**_ **= .105**							
**BLOCK 2**							
Hostility			− 0.03	− 0.91	0.37	− 0.03	[− 0.001, 0.000]
**F (1, 1022) = 0.82,** ***p*** **= 0.37, i**^**2**^ **= 0.116,** ***R***^**2**^_**adj**_ **= 0.104**							
**BLOCK 3**							
Hostility*sex			− 0.08	− 2.78	0.006*	− 0.08	[− 0.002, − 0.0003]
**F(1,102) = 7.72,** ***p*** **= 0.007,** ***R***^**2**^ **= 0.122,** ***R***^**2**^_**adj**_ **= 0.110**							
**Regression 2**							
**BLOCK 2**							
Defensiveness			0.03	0.84	0.40	0.03	[− 0.001, 0.001]
**F (1, 1022) = 0.71,** ***p*** **= 0.40,** ***R***^**2**^ **= 0.116,** ***R***^**2**^_**adj**_ **= 0.104**							
**BLOCK 3**							
Defensiveness*CAD status			− 0.06	− 1.98	0.049*	− 0.058	[− 0.004, − 0.000]
**F(1,1021) = 3.90,** ***p*** **= 0.007,** ***R*** **= 0.119,** ***R***^**2**^_**adj**_ **= 0.107**							

*CI*: confidence interval

CAD status (dichotomized)= with (1) or without CAD (2); Employment status (dichotomized) = retired or non-employed (1), full-time or part-time (2). History of CVD risk (dichotomized) = absence (0) or presence (1) of hypertension (and/or) diabetes (and/or) hypercholesterolemia and CVD agents (e.g., dyslipidemic, antihypertensive agents, insulin, oral hyperglycemics)

*p < .05

#: Number of

**Fig. 1:**
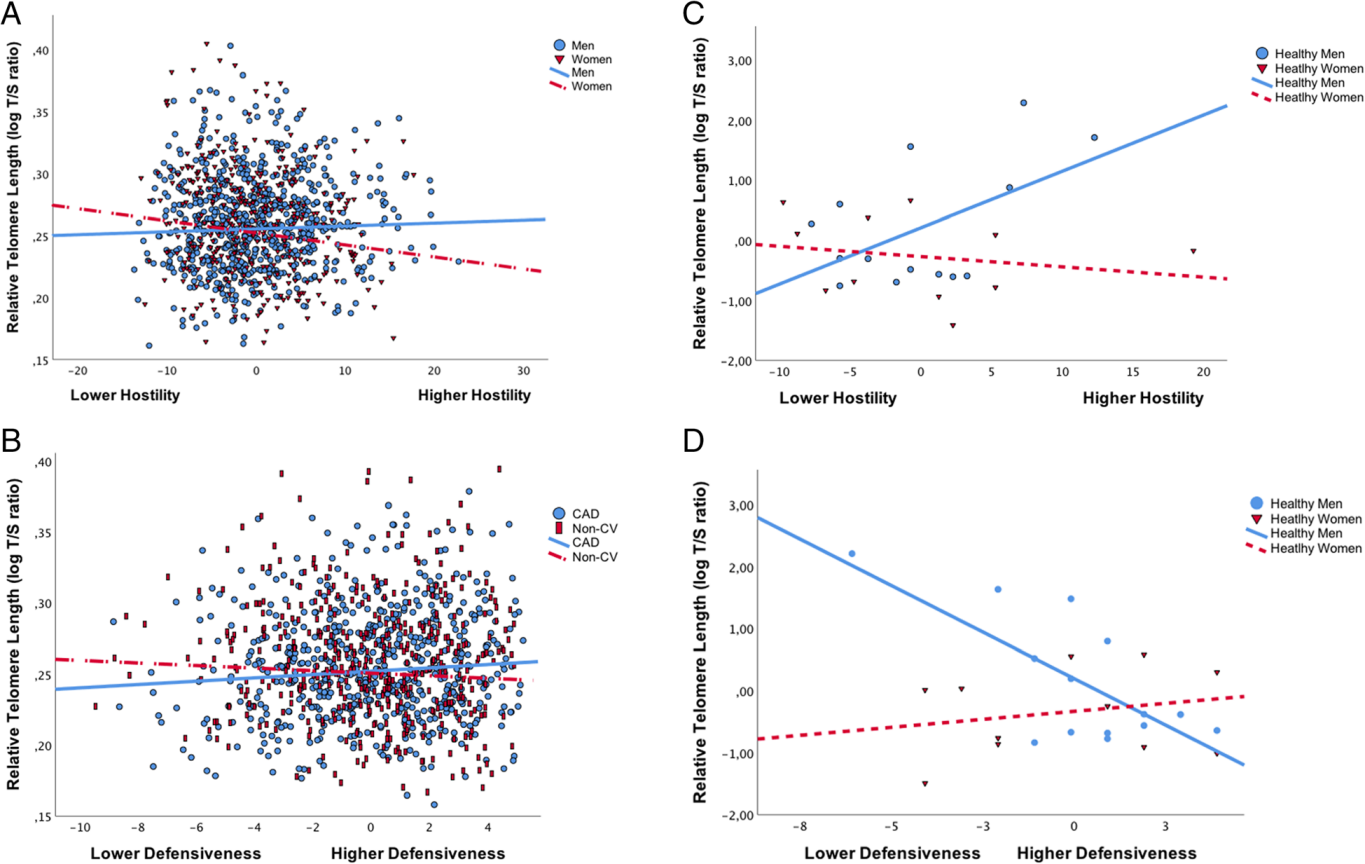
**a** The relation between hostility and TL is moderated by sex in participants with CAD or other chronic illnesses (*β* = − .08, *t* = − 2.78, *p* = .006). Greater hostility was associated with shorter TL in women (*β* = − .001, *t* = − 2.82, *p* = .005), but not in men (*β* = .001, *t* = .78, *p* = .43). **b** Among patients, the relationship between defensiveness and TL is moderated by CAD status (*β* = − .06, *t* = − 1.98, *p* = .049). Greater defensiveness tended to be associated with longer TL among individuals with CAD (*β* = .001, *t* = 1.93, *p* = .06), but not among patients with non-CV illnesses (*β* = − .001, *t* = − .93, *p* = .35). **c** In healthy individuals, the sex by hostility interaction was significant. Greater hostility was associated with significantly longer TL among men (*β* = .09, *t* = 2.37, *p* = .028), but not in women (*β* = − .02, *t* = − .54, *p* = .59). **d** The relation between defensiveness and TL is moderated by sex in healthy participants. Greater defensiveness was associated with shorter TL in healthy men (*β* = − .30, *t* = − 3.56, *p* = .002), but not in women (*β* = .05, *t* = .66, *p* = .52). Note: Relative telomere length: residuals adjusting for covariates found in respective hierarchical regressions were used to create the jittered scatterplots. Interaction terms in the main analyses were created from centred variables

#### *Defensiveness*

CAD status moderated the relation between defensiveness and TL. More specifically, greater defensiveness tended to be associated with longer TL among individuals with CAD (*β* = .001, *t* = 1.93, *p* = .05), but not among patients with non-CV illnesses (*β* = − .001, *t* = − .93, *p* = .35).

### Analyses in healthy individuals (Table 4, Fig. 1)

#### Hostility

The sex by hostility interaction was significant. Among healthy men only did TL appear to be longer in those with greater hostility (*β* = .09, *t* = 2.37, *p* = .028).

**Table 4:** Results of the hierarchical regression analysis for hostility and defensiveness predicting telomere length in healthy individuals (*n* = 25)

	*β*	*t*	*p*	Semipartial *r*	95% CI
**BLOCK 1**					
Sex	− 0.27	− 1.33	0.20	− 0.27	[− 1.23, 0.27]
**F(1,24) = 1.77,** ***p*** **= 0.20,** ***R***^**2**^ **= 0.071**					
**BLOCK 2**					
Hostility	0.20	0.97	0.34	0.20	[− 0.03, 0.08]
**F(2, 24) = 1.35,** ***p*** **= 0.28,** ***R***^**2**^ **= 0.110**					
**BLOCK 3**					
Hostility*sex	− 0.43	− 2.19	0.049*	− 0.41	[− 0.22, − 0.01]
**F(3, 24) = 2.44,** ***p*** **= 0.08,** ***R***^**2**^ **= 0.275**					
**BLOCK 2**					
Defensiveness	− 0.32	− 1.65	0.11	− 0.32	[− 0.25, 0.03]
**F(2,24) = 2.31,** ***p*** **= 0.12,** ***R***^**2**^ **= 0.174**					
**BLOCK 3**					
Defensiveness*sex	0.52	3.06	0.006**	0.51	[0.11, 0.59]
**F(3,24) = 5.25,** ***p*** **= 0.007,** ***R***^**2**^ **= 0.428**					

*CI*: confidence interval

Residualized telomere length controlling for age, body mass index, years of education, and employment status

*p < .05

#### Defensiveness

The sex by defensiveness interaction was significant. Shorter TL were observed in the healthy men who were more defensive (*β* = − .30, *t* = − 3.56, *p* = .002).

## Discussion

This cross-sectional study examined the relation between TL and two personality traits and whether these associations differed as a function of CAD status and/or sex, controlling for sociodemographic, behavioural, and medical risk factors. Sex and health status were found to moderate the relationship between hostility, defensiveness, and TL. More specifically, greater hostility was associated with significantly shorter TL in women (independent of health status) but with longer TL among healthy men, while greater defensiveness was associated with longer TL in individuals with CAD, but with shorter TL in healthy men.

The sex differential observed in the relation between hostility and TL was somewhat surprising. Indeed, we found robust evidence for shorter TL in more hostile as compared to less hostile women, but this was not observed in the men. In contrast, two previous studies had observed such results in men more particularly. One sample included predominantly male U.S. veterans with various medical conditions (including heart disease) [31], while the other was comprised of a similar number of relatively healthy older British men and women [29]. Conversely and consistent with our current findings in the healthy men, we had previously reported a positive association between hostility (and increases in hostility over time) and TL in a sample of healthy adult men and women [30]. The reasons for these discrepant results across studies may relate in part to methodological differences. For example, our previous [30] and current sample were more heterogeneous compared to that of the Whitehall-II investigation [29]. This reduced variability in the Whitehall-II investigation may explain why they showed no association between TL and chronological age as opposed to both our studies which showed the expected negative association. Although our current sample was similar in age to that of Whitehall-II investigation, it suffered from a wide range of health issues, including CAD. In Watkins’ study [30], TL was isolated from saliva, which is more sensitive to environmental confounds as compared to blood. Furthermore, the six items of the SCL-90-R used in Watkins’ study encompassed only hostility’s affective and behavioural symptoms (aggressive urges and impulses) over the last month, as opposed to its trait component as depicted in the Cook–Medley’s Hostility Scale. At this time, conclusions regarding the associations of hostility with TL remain unclear, given mixed results from one sample to another. Though men are more commonly known to be at risk for adverse effects of trait hostility on health, there has been growing evidence of its impact on illness (CAD) severity in women [56, 57], and our robust findings contribute to the body of knowledge on the potential impact of trait hostility on women’s health in particular.

Health status influenced not only the relation between TL and age, but also the relation between TL and defensiveness in the current study. Greater defensiveness was associated with longer TL among individuals with CAD but not among those with other non-CVD illnesses. Moreover, it was related to shorter TL in healthy men. We had previously reported shorter TL among men and women who were more defensive in a study involving healthy participants [30]. Findings by Shoormans and colleagues [58] may provide additional insight into the mixed results regarding TL and personality traits. They found that while those with Type D personality (a construct related to defensiveness) had shorter TL at study onset, they were also *less* likely to show TL shortening over a period of 6 years compared to those without Type D. Protection from TL loss over time may have occurred via activation of telomerase [59]. Relatedly, initial TL has been shown to be the greatest predictor of TL change over time, with relatively long telomeres tending to shorten over time, while relatively short telomeres tending to lengthen over time, possibly due to these reparative mechanisms [60]. Given the shorter TL of patients with CAD, a similar process of telomerase activation may have occurred, in essence altering the observed relations between TL and defensiveness in this study. Whether similar processes influenced the longer telomeres observed among the more hostile healthy men in this and our previous study [30] remains unclear.

Different mechanisms have been suggested to underlie the negative association of psychological risk factors with TL, including alterations in health behaviours, as well as metabolic and inflammatory processes. Importantly, we and others have previously shown that hostility and defensiveness are associated (particularly in women) with disruptions in metabolic and inflammatory activity, with increased oxidative DNA damage [10–13, 15, 61, 62], and poorer health habits such as physical inactivity and smoking [63]. Although these physiological processes and health behaviours mentioned have been associated with shorter TL [26, 64], controlling for these potential mediating or confounding factors did not alter the pattern of results in the current investigation.

### Strengths and limitations

While the variance explained by all variables, except age, was low, the findings regarding the personality traits were robust and independent of a large number of behavioural, sociodemographic, and medical variables. Moreover, controlling for symptoms of depression, anxiety, and stress did not alter the results (data not shown). The clinical significance of shorter TL in women with greater hostility compared to their low hostile counterparts in the current study remains to be evaluated. However, converting T/S ratio to base pairs (bp) indicates a 133 bp difference, suggestive of a 1–4 year biological difference (based on telomere attrition of 30–100 base pairs per year )[65–67].

Additional strengths of this study included a large sample size, use of validated questionnaires, and purposeful recruitment of as large a number of women as possible to examine sex differences. Findings differed significantly between men and women, which reinforces the importance of evaluating sex differences in this field of research. CAD status was also strictly defined and verified via medical files. Moreover, qPCR analyses were carefully controlled and assayed in batch in triplicate, reducing potential measurement errors.

Nonetheless, a few factors limit the conclusions that can be drawn from the current results. The cross-sectional design of our study limits any conclusions regarding causality. Furthermore, given that hostility and defensiveness increase risk for premature mortality, those most susceptible to the effects of these personality traits may have already passed away, contributing to our unexpected results (e.g., greater defensiveness associated with longer TL in CAD participants). Moreover, the sample consisted of mostly Francophone Canadian Caucasians which may limit the generalizability of our findings to other populations. Indeed, there is evidence for differential patterns of association of TL with biological parameters (such as blood cell count) as a function of geographical location [68, 69], which may reflect genetic differences [70], as well as differences in life exposures, access to healthcare, and/or coping resources [71]. Furthermore, recent data suggests TL differs as a function of blood leukocyte composition, with longer TL observed in blood samples with higher proportions of CD8+ T cells and B cells [72]. Notably, blood leukocyte composition changes from childhood to adulthood and differs as a function of sex and health status [66, 73–75], which may contribute to some of the unexpected findings observed here and in previous investigations. Future research examining TL should ideally control for potential leukocyte cell subtype differences. Finally, given the small sample size of the healthy group, it is possible that results concerning them may reflect Type 1 errors and are likely overfit. This, however, appears unlikely in light of the effect sizes observed in the correlations, the *p* values of the interactions, and the fact that results replicated those of a previous study in a healthy sample. Nonetheless, any results concerning them require replication in a larger sample.

### Perspectives and significance

Hostility and defensiveness were associated with altered TL among middle-aged and older individuals though the direction of effect appears to depend on their sex and/or health status. Though the clinical relevance of these findings remains to be established, these traits reflect a relatively enduring pattern of intrapersonal and interpersonal conflict, which may not only be detrimental to one’s mental health and quality of life, but also to their cellular aging processes. Moreover, whether psychological interventions targeting hostility and/or defensiveness can protect from premature aging remains to be properly evaluated, though small-scale studies suggest that mindfulness, for example, may improve TL and telomerase activity [76].

## Data Availability

The data that support the findings of this study are available from the corresponding author upon reasonable request.

## Abbreviations

TL: Telomere Length
CAD: Coronary artery disease
Non-CVD: Participants with non-cardiovascular illness
CMHo-T: 39-Item Cook–Medley Hostility Inventory
MCSD: 14-Item Marlowe-Crowne Social Desirability Scale
CI: Confidence interval
BMI: Body mass index
qPCR: Quantitative polymerase chain reaction

## References

[1] Smith TW, Glazer K, Ruiz JM, Gallo LC (2004). Hostility, anger, aggressiveness, and coronary heart disease: an interpersonal perspective on personality, emotion, and health. J Pers.

[2] Bunde J, Suls J (2006). A quantitative analysis of the relationship between the Cook-Medley hostility scale and traditional coronary artery disease risk factors. Health Psychol.

[3] Chida Y, Steptoe A (2009). The association of anger and hostility with future coronary heart disease: a meta-analytic review of prospective evidence. J Am Coll Cardiol.

[4] Klabbers G, Bosma H, van den Akker M, Kempen GI, van Eijk JT. Cognitive hostility predicts all-cause mortality irrespective of behavioural risk at late middle and older age. The European Journal of Public Health 2013; 23(4):701-705. 10.1093/eurpub/cks060 22683771

[5] Crowne DP, Marlowe D (1960). A new scale of social desirability independent of psychopathology. J Consult Psychol.

[6] Denollet J, Pedersen SS, Daemen J, De Jaegere P, Serruys PW, Van Domburg RT (2008). Reduced positive affect (anhedonia) predicts major clinical events following implantation of coronary-artery stents. J Intern Med.

[7] Mund M, Mitte K (2012). The costs of repression: a meta-analysis on the relation between repressive coping and somatic diseases. Health Psychol.

[8] Van’t Riet J, Ruiter RA (2013). Defensive reactions to health-promoting information: an overview and implications for future research. Health Psychology Review.

[9] Garofalo C, Velotti P, Zavattini GC, Kosson DS (2017). Emotion dysregulation and interpersonal problems: the role of defensiveness. Personal Individ Differ.

[10] D'Antono B, Moskowitz DS, Nigam A (2013). The metabolic costs of hostility in healthy adult men and women: cross-sectional and prospective analyses. J Psychosom Res.

[11] Lévesque K, Bureau S, Moskowitz DS, Tardif JC, Lavoie J, Dupuis G (2009). Defensiveness and metabolic syndrome: impact of sex and age. Biol Psychol.

[12] Lévesque K, Moskowitz DS, Tardif JC, Dupuis G, D'Antono B (2010). Physiological stress responses in defensive individuals: age and sex matter. Psychophysiology.

[13] Demarble JB, Moskowitz DS, Tardif JC, D'Antono B (2014). The relation between hostility and concurrent levels of inflammation is sex, age, and measure dependent. J Psychosom Res.

[14] Murdock KW, LeRoy AS, Fagundes CP (2017). Trait hostility and cortisol sensitivity following a stressor: the moderating role of stress-induced heart rate variability. Psychoneuroendocrinology.

[15] Guerrero Rodríguez C, Palmero Cantero F, Gómez-Íñiguez C (2018). Blood pressure responses of defensive hostile women when facing a real stress task. Psychol Health.

[16] Sahoo S, Padhy SK, Padhee B, Singla N, Sarkar S (2018). Role of personality in cardiovascular diseases: an issue that needs to be focused too!. Indian Heart J.

[17] Lin J, Epel E, Blackburn E (2012). Telomeres and lifestyle factors: roles in cellular aging. Mutation Research/Fundamental and Molecular Mechanisms of Mutagenesis.

[18] Srinivas N, Rachakonda S, Kumar R (2020). Telomeres and telomere length: a general overview. Cancers.

[19] Brouilette S, Singh RK, Thompson JR, Goodall AH, Samani NJ (2003). White cell telomere length and risk of premature myocardial infarction. Arterioscler Thromb Vasc Biol.

[20] Bhattacharyya J, Mihara K, Bhattacharjee D, Mukherjee M (2017). Telomere length as a potential biomarker of coronary artery disease. Indian J Med Res.

[21] Goglin SE, Goglin SE, Farzaneh-Far R, Epel ES, Lin J, Blackburn EH (2016). Change in leukocyte telomere length predicts mortality in patients with stable coronary heart disease from the heart and soul study. PLoS One.

[22] Haycock PC, Heydon EE, Kaptoge S, Butterworth AS, Thompson A, Willeit P (2014). Leucocyte telomere length and risk of cardiovascular disease: systematic review and meta-analysis. Bmj.

[23] Révész D, Verhoeven JE, Picard M, Lin J, Sidney S, Epel ES (2018). Associations between cellular aging markers and metabolic syndrome: findings from the CARDIA study. The Journal of Clinical Endocrinology & Metabolism.

[24] Tellechea ML, Pirola CJ (2017). The impact of hypertension on leukocyte telomere length: a systematic review and meta-analysis of human studies. J Hum Hypertens.

[25] Wang J, Dong X, Cao L, Sun Y, Qiu Y, Zhang Y (2016). Association between telomere length and diabetes mellitus: a meta-analysis. J Int Med Res.

[26] Astuti Y, Wardhana A, Watkins J, Wulaningsih W (2017). Cigarette smoking and telomere length: a systematic review of 84 studies and meta-analysis. Environ Res.

[27] Mathur MB, Epel E, Kind S, Desai M, Parks CG, Sandler DP (2016). Perceived stress and telomere length: a systematic review, meta-analysis, and methodologic considerations for advancing the field. Brain Behav Immun.

[28] Lin PY, Huang YC, Hung CF (2016). Shortened telomere length in patients with depression: a meta-analytic study. J Psychiatr Res.

[29] Brydon L, Lin J, Butcher L, Hamer M, Erusalimsky JD, Blackburn EH (2012). Hostility and cellular aging in men from the Whitehall II cohort. Biol Psychiatry.

[30] Starnino L. Busque L, Tardif JC, D’ Antono B. Psychological profiles in the prediction of leukocyte telomere length in healthy individuals. PloS one: 2016. 10.1371/journal.pone.0165482 PMC5082938 27788238

[31] Watkins LE, Harpaz-Rotem I, Sippel LM, Krystal JH, Southwick SM, Pietrzak RH (2016). Hostility and telomere shortening among US military veterans: results from the National Health and resilience in veterans study. Psychoneuroendocrinology.

[32] Zalli A, Carvalho LA, Lin J, Hamer M, Erusalimsky JD, Blackburn EH (2014). Shorter telomeres with high telomerase activity are associated with raised allostatic load and impoverished psychosocial resources. Proc Natl Acad Sci.

[33] Gardner M, Bann D, Wiley L, Cooper R, Hardy R, Nitsch D (2014). Gender and telomere length: systematic review and meta-analysis. Exp Gerontol.

[34] Dalgård C, Benetos A, Verhulst S, Labat C, Kark JD, Christensen K (2015). Leukocyte telomere length dynamics in women and men: menopause vs age effects. Int J Epidemiol.

[35] Denson TF, O’Dean SM, Blake KR, Beames JR (2018). Aggression in women: behavior, brain and hormones. Front Behav Neurosci.

[36] Barnett RC, Baruch GK. Social roles, gender, and psychological distress*.* In R. C. Barnett, L Biener, & G K Baruch (Eds.), gender and stress 1987; p. 122–143. Free Press.

[37] Christov-Moore L, Simpson EA, Coudé G, Grigaityte K, Iacoboni M, Ferrari PF (2014). Empathy: gender effects in brain and behavior. Neurosci Biobehav Rev.

[38] Chung J, Monroe GS (2003). Exploring social desirability bias. J Bus Ethics.

[39] Gentile C, Ditto B, Deschamps A, D’Antono B (2019). Parasympathetic response patterns are associated with metabolic syndrome among older women but not men. Ann Behav Med.

[40] Cook WW, Medley DM (1954). Proposed hostility and pharisaic-virtue scales for the MMPI. J Appl Psychol.

[41] Barefoot JC, Peterson BL, Dahlstrom WG, Siegler IC, Anderson NB, Williams RB (1991). Hostility patterns and health implications: correlates of Cook-Medley hostility scale scores in a national survey. Health Psychol.

[42] Boyle SH, Williams RB, Mark DB, Brummett BH, Siegler IC, Helms MJ (2004). Hostility as a predictor of survival in patients with coronary artery disease. Psychosom Med.

[43] Han K, Weed NC, Calhoun RF, Butcher JN (1995). Psychometric characteristics of the MMPI-2 Cook-Medley hostility scale. J Pers Assess.

[44] Loo R, Thorpe K (2000). Confirmatory factor analyses of the full and short versions of the Marlowe-Crowne social desirability scale. J Soc Psychol.

[45] Ii AZ, Sipps GJ (1985). Cross-validation of a short form of the Marlowe-Crowne social desirability scale. J Clin Psychol.

[46] Crowne DP, Marlowe D (1964). The approval motive: studies in evaluative dependence.

[47] Pauls CA, Stemmler G (2003). Substance and bias in social desirability responding. Personal Individ Differ.

[48] Epel ES, Blackburn EH, Lin J, Dhabhar FS, Adler NE, Morrow JD (2004). Accelerated telomere shortening in response to life stress. Proc Natl Acad Sci.

[49] Cawthon RM (2002). Telomere measurement by quantitative PCR. Nucleic Acids Res.

[50] Rudolf MC, Walker J, Cole TJ (2007). What is the best way to measure waist circumference?. Int J Pediatr Obes.

[51] Benetos A, Gardner JP, Zureik M, Labat C, Xiaobin L, Adamopoulos C (2004). Short telomeres are associated with increased carotid atherosclerosis in hypertensive subjects. Hypertension.

[52] Lenhard W (2016). Lenhard a.

[53] Hayes AF. Introduction to mediation, moderation, and conditional process analysis: a regression-based approach: American Psychologist; 2017.

[54] Rosnow RL, Rosenthal R (1989). Statistical procedures and the justification of knowledge in psychological science. Am Psychol.

[55] Winer BJ (1962). Statistical principles in experimental design.

[56] Shah AJ, Ghasemzadeh N, Zaragoza-Macias E, Patel R, Eapen DJ, Neeland IJ (2014). Sex and age differences in the association of depression with obstructive coronary artery disease and adverse cardiovascular events. J Am Heart Assoc.

[57] Pimple P, Lima BB, Hammadah M, Wilmot K, Ramadan R, Levantsevych O (2019). Psychological distress and subsequent cardiovascular events in individuals with coronary artery disease. J Am Heart Assoc.

[58] Schoormans D, Verhoeven JE, Denollet J, van de Poll-Franse L, Penninx BW (2018). Leukocyte telomere length and personality: associations with the big five and type D personality traits. Psychol Med.

[59] Geserick C, Blasco MA (2006). Novel roles for telomerase in aging. Mech Ageing Dev.

[60] Epel ES, Merkin SS, Cawthon R, Blackburn EH, Adler NE, Pletcher MJ (2009). The rate of leukocyte telomere shortening predicts mortality from cardiovascular disease in elderly men. Aging (Albany NY).

[61] Irie M, Asami S, Nagata S, Ikeda M, Miyata M, Kasai H (2001). Psychosocial factors as a potential trigger of oxidative DNA damage in human leukocytes. Jpn J Cancer Res.

[62] Elovainio M, Merjonen P, Pulkki-Råback L, Kivimäki M, Jokela M, Mattson N (2011). Hostility, metabolic syndrome, inflammation and cardiac control in young adults: the young Finns study. Biol Psychol.

[63] Wong JM, Na B, Regan MC, Whooley MA (2013). Hostility, health behaviors, and risk of recurrent events in patients with stable coronary heart disease: findings from the heart and soul study. J Am Heart Assoc.

[64] Révész D, Milaneschi Y, Verhoeven JE, Lin J, Penninx BW (2015). Longitudinal associations between metabolic syndrome components and telomere shortening. The Journal of Clinical Endocrinology & Metabolism.

[65] Müezzinler A, Zaineddin AK, Brenner H (2013). A systematic review of leukocyte telomere length and age in adults. Ageing Res Rev.

[66] Aviv A, Valdes AM, Spector TD (2006). Human telomere biology: pitfalls of moving from the laboratory to epidemiology. Int J Epidemiol.

[67] Harris SE, Marioni RE, Martin-Ruiz C, Pattie A, Gow AJ, Cox SR, Corley J, Von Zglinicki T, Starr JM, Deary IJ (2016). Longitudinal telomere length shortening and cognitive and physical decline in later life: the Lothian birth cohorts 1936 and 1921. Mech Ageing Dev.

[68] De Meyer T, De Buyzere ML, Langlois M, Rietzschel ER, Cassiman P, De Bacquer D (2008). Lower red blood cell counts in middle-aged subjects with shorter peripheral blood leukocyte telomere length. Aging Cell.

[69] Mollica L, Fleury I, Belisle C, Provost S, Roy DC, Busque L (2009). No association between telomere length and blood cell counts in elderly individuals. Journals of Gerontology Series A: Biomedical Sciences and Medical Sciences.

[70] Kim S, Parks CG, Xu Z, Carswell G, DeRoo LA, Sandler DP, Taylor JA (2012). Association between genetic variants in DNA and histone methylation and telomere length. PLoS One.

[71] Geronimus AT, Pearson JA, Linnenbringer E, Schulz AJ, Reyes AG, Epel ES (2015). Race-ethnicity, poverty, urban stressors, and telomere length in a Detroit community-based sample. J Health Soc Behav.

[72] Kresovich JK, Parks CG, Sandler DP, Weinberg CR, Taylor JA (2020). The role of blood cell composition in epidemiologic studies of telomeres. Epidemiology.

[73] Gomez-Sanchez L, Garcia-Ortiz L, Recio-Rodriguez JI, Patino-Alonso MC, Agudo-Conde C, Rigo F, Ramos R, Marti R, Gomez-Marcos MA, MARK Group (2015). Leukocyte subtype counts and its association with vascular structure and function in adults with intermediate cardiovascular risk. MARK study. PLoS One.

[74] Kolbus D, Ljungcrantz I, Andersson L, Hedblad B, Fredrikson GN, Björkbacka H (2013). Association between CD 8+ T-cell subsets and cardiovascular disease. J Intern Med.

[75] Lin Y, Damjanovic A, Metter EJ, Nguyen H, Truong T, Najarro K (2015). Age-associated telomere attrition of lymphocytes in vivo is co-ordinated with changes in telomerase activity, composition of lymphocyte subsets and health conditions. Clin Sci.

[76] Schutte NS, Malouff JM (2014). A meta-analytic review of the effects of mindfulness meditation on telomerase activity. Psychoneuroendocrinology.

